# On Diseases of the Heart

**Published:** 1831-04-01

**Authors:** 


### DR. ELLIOTSON ON DISEASES OF THE HEART.

[Lecture the Third and Last.]

### ON DISEASES OF THE SUBSTANCE OF THE HEART.

WE frequently observe the parietes of the heart thickened or dilated, during or subsequent to attacks of acute rheumatism.

"The increase of the heart's substance, is, no less than the diseases of the valves, I am convinced, with Bouillaud, Bertin, Andral, and others, generally inflammatory. I have witnessed, after acute rheumatism, a constant and severe pain in the region of the heart, palpitation, and at length death; and found the left ventricle thickened, without any other morbid appearance in the organ, and here, no less than when the ordinary anatomical results of inflammation were visible, it was impossible not to consider the change inflammatory. On comparing an hypertrophied left ventricle with a healthy specimen, M. Chevalier, a pupil of Vauquelin's, found, under the microscope, that its fibres were redder; and, a portion of each being steeped in a separate quantity of distilled water, the hypertrophied portion reddened the water more than the other, and when taken out was still the redder of the two. On being put into boiling alcohol, it proved to contain less fatty matter. Indeed an hypertrophied heart, if undilated, is always in the affected portions redder and firmer than usual, and its coronary arteries are enlarged, sometimes enormously. Besides, as hypertrophy is a common accompaniment of chronic inflammation of the outer or inner membrane of the heart, no doubt can exist upon the subject. Indeed its essence, being excessive nutritive action, cannot but be in some measure inflammatory. It is unquestionably produced in some cases by obstruction in the opening leading from the cavity whose walls are thickened, the difficulty giving rise to evident great muscular effects, and consequently to augmented circulation, of the organ. But even here it may be to some degree absolutely inflammatory, because the lining membrane of the cavity has generally been in an inflamed state, which in truth was the cause of the valvular obstruction.

Hypertrophy of the left ventricle is often found with disease of the lining membrane of the aorta; sometimes coexistent with the true and false aneurism of the aorta, but not unfrequently is the sole diseased appearance.

Hypertrophy is frequently attended with dilatation, and more so perhaps when an obstruction to the egress of the blood from the diseased compartment exists. Dilatation often occurs alone, especially in the auricles, from this cause. But, like hypertrophy, it is sometimes the only morbid circumstance discernible, and therefore not so easily to be explained. The dilated portion, we shall find, is occasionally thin and soft, and the reason of the dilatation is then evident.

Hypertrophy is considered by Bertin, accordingly as the compartment of the heart is simply thickened, without any change in the dimensions of the cavity; as it retains its natural thickness, but is dilated, in which case there must be an hypertrophy or preternatural quantity of substance, or it would have become thinner; and thirdly, as it is not merely thickened, but its cavity diminished. He designates these three forms simple hypertrophy, eccentric hypertrophy, and concentric hypertrophy.

The second variety, eccentric hypertrophy, or increase of substance and dilatation united, was described by Senac, Morgagni and Lancisi, and denominated by Corvisart, active aneurism of the heart. It is the most common of the three, and the excessive deposition must have taken place in the direction of the surface. The first variety, simple hypertrophy, in which the dimensions of the cavity are unchanged, and the deposition takes place also in the direction of the surface; and the third, the concentric, in which the cavity is diminished, and the deposition must have occurred in a vertical direction—at a right angle with the surface, were first described in 1811, by Bertin. In the concentric, the cavity is sometimes so small that it will scarcely contain the shell of an almond.

Every heart with thick parietes and a small cavity, however, is not perhaps an example of concentric hypertrophy. A violent contraction at death might probably produce these effects. But in such a case, the bulk of the organ would be proportionately lessened. To be justified, therefore, in declaring the existence of concentric hypertrophy, not only the parietes should be thick and the cavity small, but the bulk natural.

In the simple and concentric hypertrophy of the left ventricle, the thickness is sometimes double, or even triple, what is natural: the natural thickness of the walls of the left ventricle are about half an inch.

The thickening of the left ventricle is generally greatest at the base, in the first and second varieties, and lessens towards the apex. In the concentric, there is usually as much thickening at the apex as at the base.

The septum of the ventricles, and even the columnæ carneæ, participate in the increase of substance.

The thickening is sometimes unequal in different parts, both in the case of the left ventricle and of any other part that may be hypertrophied.

The right ventricle, when not participating in the disease, is comparatively so small, and reaches so short a way down, that it looks like a mere appendage, or as if included in the walls of the heart.

The left ventricle is the most usual seat of hypertrophy. When the disease occurs in the right, the thickening is seldom, if ever, so great, and is generally more uniform: but the columnæ carneæ, it is said, are generally more enlarged in proportion.

Sometimes both ventricles are hypertrophied; sometimes one is hypertrophied, in one or other of the three varieties of the affection, while its fellow is only dilated.

Hypertrophy rarely affects the auricles; and still more rarely unless other disease exists in the heart. It is generally united with dilatation of the auricle. Laennec and many others never witnessed an instance of pure hypertrophy of an auricle.

If the walls of all the cavities of the heart are affected with hypertrophy and dilatation, or even both ventricles only, the heart may acquire an enormous size. Under these circumstances its form usually becomes rounded, its apex being effaced, and it lies amost transversely in the chest." 26.

In dilatation of the heart, the natural thickness of the parietes may be retained, or their thickness may be augmented; but there is a third variety of dilatation—the thickness may be diminished, and this used to be called passive aneurism. "The first of these varieties,—that in which the dilated parietes retain their natural thickness, appears to me, I confess, as true an hypertrophy as the second in which the thickness is increased, because dilatation would inevitably occasion attenuation, were not an addition of substance made." Two or even three of these varieties may exist in the parietes of the same cavity. Laennec regards original disproportion of the cavities as the most powerful cause of dilatation; but a very common cause undoubtedly is obstruction to the progress of the blood from the cavity, and the source of this obstruction may be seated in another cavity—in one farther forward relatively to the blood's course. The auricle is frequently dilated and not the ventricle, when there is an impediment at the opening of the aortic or pulmonary artery:—and, when the difficulty is at the aortic opening, it is curious that it is sometimes the right, not the left auricle that is dilated. The obstacle may arise from a diminution of an opening, or from diminution of another cavity, as of the left ventricle. A permanent patency of the auriculo-ventricular opening, by which too much blood attempts to rush back into the auricle, will create marked dilatation of it. The auricles, from their thinness, are more subject to dilatation than the ventricles—and the right is more so than the left, from the frequency of obstruction in the lungs. There is reason to believe that the right auricle often becomes dilated in the dying state, especially when death is lingering, from impediment to the passage of blood through the lungs. Let us now inquire into the signs of hypertrophy of the heart, as sketched by our author.

"When the heart is hypertrophied, an unusually strong impulse is given in the situation of the part affected, and the sound usually heard there is diminished,—becomes a sort of murmur, and in severe cases it is not heard at all. The contraction takes place more slowly than in the healthy state, and, in the hypertrophy of the ventricles, little or no interval of repose occurs after the auricular action. When the heart is dilated, the reverse happens:—there is far less than the usual impulse in the situation of the part affected, and the sound usually heard there is augmented and is not merely louder but clearer than natural. In dilatation of the ventricles, their action takes place more rapidly than in the healthy state, and little or no interval of repose occurs equally as in hypertrophy.

In hypertrophy of the left ventricle,—its most common seat, a strong impulse is felt between the cartilages of the fifth and sixth left ribs, and the action of the auricles is very short. In hypertrophy of the right ventricle, this strong impulse is felt at the lower part of the sternum.

If the difference is considerable, it is easily discovered by the greater force with which the stethoscope is repelled in the affected side, when applied to the cartilages and the sternum in succession. Where the difference is less, it may, I think, be more easily ascertained, perhaps, by examining both sides at once in placing the hand longitudinally over the cardiac region, when the stronger impulse against the fingers lying over the left or the right half of the region will presently be noticed. In health, the impulse in both halves is equal. Should hypertrophy affect both ventricles at once, the excess of impulse in both halves will determine its existence in both, and the equality or difference of the morbid impulse will demonstrate its equality or difference in the two ventricles.

Hypertrophy of the auricles must be very considerable to afford an impulse. This, however, will be felt on the right or left superior half of the cardiac region, according to the situation of the hypertrophy.

The situation of the loud and clear sound of dilatation equally agrees with the seat of the dilatation.

It is wonderful how accurately we can determine the seat and nature of these diseases. If due attention is given and the symptoms carefully investigated, a physician may pronounce with certainty, frequently even at the first visit, that such a ventricle or both are hypertrophied or dilated, or the two in opposite states; that such an auricle or both are hypertrophied or dilated, or the two in opposite states,—no less than that an opening in a particular cavity is deranged: and an examination after death will verify his assertions. Laennec considers these results of auscultation, when well marked, infallible, and so I believe them. A good auscultator will never object, after making a full investigation, to write down his opinion and deliver it to another for the purpose of being read at the autopsy.

When hypertrophy and dilatation coexist in the walls of a cavity, the two sets of auscultatory signs are blended, and those of hypertrophy or of dilatation predominate accordingly as either state predominates.

In general the hypertrophy exceeds the dilatation; for, not only do we continually see an increase of thickness with dilatation, but, however extensive the dilatation, if the natural thickness is undiminished, nay, even if it is diminished but not in proportion to the dilatation, hypertrophy must still exist.

In these compound cases, therefore, the sign of hypertrophy,—the preternatural impulse, is generally the more prominent phenomenon, and I own myself to have repeatedly pronounced a case to be one of hypertrophy, when the cavity was likewise to a certain degree dilated; the dilatation, however, being much inferior to the hypertrophy, and the injury to the system arising not from the dilatation but from the hypertrophy.

This combination is every day seen in the left ventricle, the great disease of which is hypertrophy, but with its hypertrophy there is generally more or less dilatation.

When the left ventricle is severely affected in this two-fold manner, its violent pulsations are felt not merely in the left lower half of the cardiac region, but also under the sternum, and even to the right of this bone. For the growth of the diseased ventricle pushes the right outwards and backwards, and occupies its place as well as the left side. But there is violent impulse equally in the left half of the cardiac region, proving the hypertrophy of the left ventricle, and there is a dull sound on striking the sternum, from the presence there of the enlarged left ventricle.

The cardiac region of the front of the chest corresponds with the lower third of the sternum, and the cartilages of the fourth, fifth, sixth and seventh ribs, thus having a right and a left half. The right or sternal half naturally sounds clear on percussion, and often even under dilatation of the right cavities: but enlargement of the heart from hypertrophy and dilatation, or the deposition of solid or fluid upon or around the organ, makes it sound dull or dead. The left or costal half of the cardiac region naturally sounds rather dull in most persons, and even dead in fat, anasarcous, and muscular persons, except at the highest—the auricular part, where some hollowness of sound is almost always heard, unless in enormous dilatation of the auricles, such as exists only with constriction, Laennec says, of the left auriculo-ventricular opening.

It is in some of these instances of combination of the two affections in the left ventricles, that we frequently see those dreadful cases of violent palpitation and of throbbings of the arteries: and for no other reason, I presume, than that, in these, hypertrophy is carried to the highest pitch; for when, notwithstanding extensive dilatation, the walls are thickened, the quantity of additional substance must be enormous.

Towards the close of even this variety of hypertrophy, the impulse is often much reduced, and the heart sometimes grows soft, so that the pulse is weak, and the morbid action of the heart recognized only in the cardiac region. The occurrence of pneumonia may also obscure the stethoscopic signs while it lasts.

A considerable dilatation of the heart necessarily causes a dead sound to be heard more extensively than usual on striking the cardiac region with the ends of the fingers, and M. Piorry assures us, that, if a hard elastic substance is interposed at the time between the chest and the fingers, such a distinctness of sound is produced, that the size of the portions of the heart immediately under the sternum and ribs may be ascertained with the greatest nicety." 27.

The auscultatory signs are the only accurate means of judging in these cases. The pulse, the respiration, the state of the veins, of the cellular membrane, of the countenance, &c. may all suffer; but the changes induced are too varied for any reliance, and may be produced by many other causes besides hypertrophy of the heart.

In hypertrophy, especially with dilatation, the pulse is frequently full, causing head-ache, and even apoplexy, if the vessels of the brain be weak or diseased. But when the opening of the mitral or aortic valves is narrowed, the pulse may be very small, while the left ventricle is hypertrophied and beats violently. In mere hypertrophy, too, it is small, if the cavity of the ventricle be diminished. It is curious that irregularity of the pulse is so common to some people, that it only becomes regular in a state of excitement or disease. In other's the irregularity of the pulse depends on disordered states of the head, but more especially of the stomach. Tendency to faintings may be a purely nervous affection, or from sympathy. Violent action of the heart and arteries, as in nervous palpitation, will be distinguished from hypertrophy, by being only temporary—and by being a general increased action of all the chambers of the heart, whereas hypertrophy almost always affects one side more than the other.

"Again the symptoms produced by a diseased heart will sometimes so remit, either from a decline of the ordinary irritation of the heart, or the cessation of some temporary irritation of another organ, as to create a doubt in the mind of any but an auscultator, that organic disease of the heart had really existed. Even the dropsy, frequently attendant upon organic disease of the heart, subsides and returns, without evident reason, or accordingly as the patient is quiet in body and in mind, and careful with respect to diet and vicissitudes of temperature, or the reverse.

Frequently, too, auscultation will disclose cardiac disease, and the symptoms may not increase for many years, so that the auscultator may be ridiculed, and no organic affection be admitted; when, from unfavourable circumstances or the progress of life, the disease may suddenly begin to augment, and dissection disclose some organic affection. Many persons die of organic cardiac disease after forty, who, for many years of their lives, had merely a little short breath with occasional palpitation, and swelling of the feet, and were told that they were free from all disease of the heart. Indeed Laennec was convinced, that congenital disproportion of the cavities of the organ was one of the great predisposing causes of its dilatation. In slight cases of the blue disease, where a small direct communication existed between the right and left sides of the heart, I have seen the symptoms, after being stationary during the whole of infancy, adolescence, and the earlier part of manhood, at once augment; from the sudden enlargement, I presume, of the preternatural opening, either through the enlargement of one of the cavities between which it formed a communication, or the occurrence of narrowness of one of the natural openings of the heart farther on." 28.

The general symptoms, however, are never overlooked by the judicious auscultator, for they are valuable auxiliaries in the diagnosis. Dr. Elliotson makes several judicious remarks on those partial aneurisms of the heart which we occasionally though rarely observe. Aneurism is a disease of arteries, never of veins—and it is remarkable that this disease has never been detected but in the left, or arterial side of the heart—and never but in the ventricle.

"This circumstance accords both with the general observation of the greater frequency of all diseases on the left than the right side of the organ, and in the ventricle than the auricle; and with the other fact of the individual disease—aneurism, being an arterial disease, or rather, I would say, a disease of parts containing decarbonised blood, for the pulmonary artery, containing black blood, has not, I believe, yet been seen the seat of aneurism.

As far as appears known, all the instances, but one, have occurred in adult males, and *arterial* aneurism occurs eight times more frequently in males than in females.

This cardiac affection is the first of those in which auscultation renders no assistance. All the cardiac diseases in which it is useful have been considered. The general symptoms which occur may well therefore be supposed, by those who know the value of auscultation, to leave the nature of the disease in doubt.

Dr. Baillie says the symptoms 'are similar to those which belong to aneurism of the arch of the aorta.' And what does Dr. Baillie enumerate as the symptoms of aneurism of the arch of the aorta? First, difficulty of breathing. But that may arise from a thousand causes in the heart, lungs, or other parts, and has been absent in this disease. Secondly, more or less of pain in the aneurismal tumour, or some other part of the chest. But here is no tumour, and pain in some other part of the chest would certainly never lead us to a knowledge of the disease, but rather to a misconception of its seat and nature. The pulse, he says, is sometimes irregular; but immediately subjoins, that 'often no irregularity can be felt in it.' The chief symptoms he pronounces to be 'a strong pulsation in the chest, commonly visible to the eye, when the chest is exposed to view.' But, again immediately subjoins, that he has felt the same kind of pulsation in other cases: for example, in adhesion of the pericardium, slight pericarditis with slight hydro-pericardium, and in ordinary morbid enlargment of the heart. Indeed no strong pulsation, except palpitation of the heart, and in most cases not even that, has occurred in this affection. The most decided characteristic of the disease, however, belonging only to aneurism, he regards as a strong pulsating external tumour when the aneurism has attained a large size. But aneurism of the heart rarely attains a large size, and I do not know that it ever produced an external tumour. And after all, a diagnosis would be required between aneurism of the heart and of the arch of the aorta.

It therefore really appears to me, and I say it with all the respect due to an amiable and industrious man, that Dr. Baillie would have done better in saying, that there were no particular symptoms of the disease, especially as he never met with more than one example of it.

In Talma, who died of strangely neglected stricture of the rectum, no symptoms had led to a suspicion of cardiac disease. In a patient of Dr. Johnson's,—General Kidd, death took place suddenly from rupture of the aneurism, without any previous symptom of its existence. The same happened in Dr. Reynaud's case. In a patient of Cruveilhier's, severe asthmatic paroxysms took place, with a sense of constriction, particularly about the heart, and a peculiar sensation in the arm-pits. These symptoms, together with hardness, fulness, and occasional intermittence of the pulse, and suffusion of the face, led to the suspicion of organic disease in the heart. But auscultation at that time was not yet cultivated. The hardness and fulness of the pulse arose from hypertrophy of the left ventricle.

In the case which supplied this preparation, the patient, a man aged thirty-two, was admitted into St. Thomas's Hospital on March 2, 1826, on account of palpitation of the heart, especially on moving or lying down; inability to lie in any other position than on his back; sudden starting up during sleep; constant dyspnœa, and cough, with a little mucous expectoration. The pulse was regular, not much quicker than natural, but exceedingly weak.

He had discharged large quantities of tape-worm by means of oil of turpentine, and suffered severely from rheumatism the preceding winter and was still complaining of it. These symptoms gave no information as to the nature of his disease; they led to a suspicion only of some affection of the heart.

I was not at that time well practised in auscultation, but found the ventricular impulse of the heart very strong all over the right half of the cardiac region; and therefore concluded that the disease existed in the right ventricle.

In two months, he became much better under rest and moderate antiphlogistic treatment, experienced less palpitation and dyspnœa, could lie on both sides, and was stronger. The ventricular action was no longer strong on the right side, but was now both loud and strong in the lower part of the left. I could thus form no opinion as to the seat or nature of the disease.

The dyspnœa after this time gradually increased for a fortnight, and he then died.

On opening the body, two aneurisms were found at the upper part of the left ventricle; but on the right ventricle was a tumour or large tubercle, the size of a pigeon's egg, with several smaller ones.

The force originally observed in the right half of the cardiac region was now explicable, although the aneurisms sprang from the left ventricle. The aneurisms, when I first saw him, were probably not large enough to occasion so considerable symptoms as the tumour on the right ventricle; but, as they increased, decided symptoms necessarily arose in the left half of the cardiac region, much exceeding those produced in the right by the tumour." 29.

Dr. E. has lately met with a case of an auricle, a part he had never before seen affected in this way. There was hypertrophy and dilatation of the left ventricle, with extreme cohesion and ossification of the mitral valve, the auriculo-ventricular opening being proportionally reduced. The sinus of the left auricle formed an aneurism of large size, with very dense layers of fibrine for its walls, which had reduced the cavity to a very small capacity. The action of the ventricles had been strong, loud, and extended, with a dull sound on percussion of the cardiac region. The pulse had been feeble and the breathing very difficult. The legs had swelled. The feebleness of the pulse, notwithstanding the strong action of the left ventricle, arose from the small quantity of blood discharged into that ventricle for delivery into the aorta.

"Hypertrophy of the heart, I have stated, appears of an inflammatory nature. With it is generally united some degree of induration. Sometimes induration exists alone, or in great proportion to the hypertrophy. I believe it is attended by palpitation, a full and rather hard pulse, and frequently by pain in the heart. One must suppose it inflammatory. On the other hand, the substance of the heart is sometimes softened, and the pulse and action of the heart are then feeble and frequent, dropsy takes place, and a disposition to fainting, and the heart is generally found also dilated, and of a deeper or paler colour than usual. This state also may be the result of inflammation, as softening of other organs beyond all question frequently is. Yet softening of the heart and all other parts, and even gangrene, probably may occur without inflammation; and sometimes when it occurs with inflammation, the inflammation may be not the cause, but only one of the circumstances of a peculiar condition.

Very acute inflammation of every part generally, I believe, induces a softened state: and, although a softened state may also result from a chronic inflammation, chronic inflammation generally causes induration. This is seen in regard to the various muscles of the body, as well as to the various internal organs.

Great as is the modern improvement in pathology by the knowledge that many diseases are essentially, or to a degree, inflammatory, which were formerly not regarded so, and great as is the improvement in medical practice by the administration of antiphlogistic measures in such cases, I am satisfied that we are now rather too prone to believe affections nothing more than inflammations, and to treat them only as such,—that many so regarded are not at all inflammatory, or have inflammation, not as a fundamental state, but merely as one circumstance among the morbid actions, or really have inflammation as a consequence, and that the hope of curing them by only antiphlogistic measures is vain, whatever relief may be effected by lessening the inflammation which accompanies them.

The deep-coloured and the pale softening of the heart, distinguished by

Laennec, occur, according to Bertin, the former as an acute, the latter as a chronic, affection. The chronic is often an attendant upon general cachexy; the acute, on pericarditis.

But, besides these chronic changes, true acute inflammation of the substance does occasionally occur.

Abscess and circumscribed ulceration are more frequently found.

The latter may commence in the lining membrane or pericardium. But, whether the ulceration be the result of an abscess of the heart, or originate in one of these membranes, it may proceed to perforation; or to such attenuation that rupture ensues, perhaps on some effort." 30.

Abundant cases of rupture of the heart are now on record, independently of ulceration. The following particulars of the death of Mr. Hayes of Charlotte Street, Fitzroy Square, a worthy surgeon, may be interesting.

"Before committing these lectures to the press, the first instance of rupture of the heart has been witnessed by me, in company with my friends Dr. Roe and Mr. Alcock. The rupture was a zigzag fissure in the front of the left ventricle, not at once discerned on bringing the heart into view, but which had filled the pericardium with blood. The patient, Mr. Hayes, a highly respectable general practitioner, in Charlotte Street, Fitzroy Square, corpulent and about sixty years of age, had suffered three attacks of pain about the præcordia with dyspepsia and vomiting, during six days, but gone out as usual in the intervals. On the morning of his decease, a fourth attack of pain, referred by him to the heart, took place before he rose in the morning, and he gave his assistant directions for preparing him a fomentation. Presently his bell rang violently, and was instantly answered; but he was found dead. The heart was very soft: the fat external to the abdomen excessive; and considerable adhesions existed among the abdominal viscera." 30.

George the Second perished suddenly by rupture of the right ventricle—which is a very unusual occurrence—as did also the Princess of Brunswick in 1730. The valves are sometimes suddenly torn, and when the rent is extensive it is probable that extreme dyspnœa and frequency of pulse are the consequence.

Our author has not read of gangrene of the heart, but it has been found soft and black, without gangrenous smell—probably from excessive congestion of blood. The induration of the heart occasionally amounts to a cartilaginous or osseous state. These changes are almost always originally seated in the lining membrane or the pericardium—or in the subjacent cellular substance. Sometimes nearly a whole cavity becomes bony. Mr. Burns declares that he once saw not only complete ossification of the pericardium, but also the ventricles ossified as firmly as the skull, except for about an inch at their apex. In that case, Dr. E. has omitted to notice that the auricles were enlarged and thickened, and, in fact, performed the office of the ventricles in carrying on the circulation. Dr. Elliotson regards ossification of the coronary arteries as sometimes the cause of those symptoms denominated angina pectoris; but properly observes that such ossifications frequently obtain without angina pectoris, and angina pectoris without them. Dr. E. has seen the substance of the heart almost entirely changed into fat. The patient had been subject to frequent attacks of syncope, palpitation, irregular and small pulse, with dyspnœa and anasarca.

"Like various parts of the body, the heart is subject to neuralgia. I had a case of this kind, where the pain was intense, affecting not only the region of the heart, but darting at the shoulders and back, sometimes dull and aching, at others, stabbing: not increased by motion or posture; unaccompanied by any stethoscopic signs.

It occurred in a middle-aged woman, at the hospital; and the symptoms were removed by iron, but afterwards, I believe, returned."* 33.

Although the greater part of the fibrinous coagula observed in the heart and great vessels are the products of death, yet some are unquestionably produced during life; for they are occasionally organic, and adhere by vessels to the substance of the heart, with corresponding symptoms during life.

"I formerly mentioned that the lining membrane of the heart and aorta was subject to inflammation, and sometimes simultaneously with the pericardium. In the case of the heart, I believe that the symptoms are increased general sound, force and frequency of action, with more or less pain or uneasiness: and when the former continue for a length of time, without the peculiar auscultatory signs of organic disease, or proportionate pain on pressure as observed in pericarditis, we certainly may infer the existence of this inflammation.

The lining membrane of the aorta is generally inflamed at the same time, and, when the affection extends along the descending portion, there is frequently a sense of smarting in the direction of the spine, and a violent pulsation in the abdomen not only of the aorta, but even of the iliacs, without any perceptible enlargement of the vessels, the existence of any tumour in its neighbourhood, or such a smallness of any of the arteries as might render it probable that the aorta was narrowed in any part and thus forced into violent pulsation.

Aortitis seems caused by all the circumstances which produce inflammatory affections of the heart, and perhaps by disease of the heart itself. Either hypertrophy of the left ventricle gives rise to inflammation of the aorta, or the converse happens, or they spring up together, for they are frequently conjoined.

Of the intensely bright redness of the inner surface of the inflamed aorta, and the impossibility of ascribing it to any thing but inflammation, I formerly spoke, as well as of the occasional effusion of fibrine. It appears as a red stain, and sometimes the middle coat appears more loaded with blood than usual.

But we far more frequently discover the chronic change of structure in this vessel. Yellow spots are seen every day: often a yellow curdy substance can be squeezed through the inner membrane under which it is deposited, the membrane having become friable and under distention giving it issue." 34.

Dr. Elliotson concludes the work with some judicious observations on aortic aneurisms; but nothing that need detain us any longer. We have now given a very full analysis of these lectures, to which some good plates are appended in illustration. The form of the work will greatly abridge its

---

* "A second case has occurred to me since the delivery of these lectures, in the person of a lady, who appears to have laboured under the disease very many years. Besides a constant aching in the lower part of the cardiac region near the sternum, are frequent stabbing pains, plunging, (to use the expression of most persons afflicted with neuralgia,) so that she is compelled to rise and walk the room almost every night. These occur during motion and rest, but most severely during motion. They shoot in all directions, and occur occasionally in distant nerves. As soon as they are over she is apparently well, and, when they are not very troublesome, sings, dances, or takes horse exercise. In the first case, the ear discovered nothing morbid in the heart: in this, a slight ventricular bellows-sound is heard. Andral mentions a dreadful case of this kind, which proved fatal, without leaving any trace of disease in the heart, or its dependencies. Précis d'Anatomie Pathologique, t. ii. page 344, sq."

circulation among the profession, and this circumstance has induced us to dedicate a greater than usual space to its contents.

